# Hypoglossal Nerve Stimulation Respiratory Lead Migration: MAUDE Database Review and Case Report

**DOI:** 10.1002/oto2.70141

**Published:** 2025-06-12

**Authors:** Emily A. Commesso, Marcus F. Paoletti, Eric J. Kezirian

**Affiliations:** ^1^ Department of Head and Neck Surgery & Communication Sciences Duke University School of Medicine Durham North Carolina USA; ^2^ Keck School of Medicine of the University of Southern California Los Angeles California USA; ^3^ Department of Head and Neck Surgery David Geffen School of Medicine at UCLA Los Angeles California USA

**Keywords:** decision tree, hypoglossal nerve stimulation, obstructive sleep apnea, pneumothorax, reoperation

## Abstract

**Objective:**

Unilateral hypoglossal nerve stimulation (HGNS) to treat obstructive sleep apnea involves implantation of a pulse generator, a respiratory sensing lead, and a stimulation lead. Complications may arise related to all components. Previous studies have presented the overall incidence of reported adverse events. The objective of this study was to provide an updated report of complications from the Food and Drug Administration's Manufacturer and User Facility Device Experience (MAUDE) database, with a focus on the respiratory sensing lead, and propose a care algorithm with two cases of sensing lead migration.

**Study Design:**

Retrospective cross‐sectional study, case report.

**Setting:**

Tertiary care center.

**Methods:**

The MAUDE database was queried for events related to the HGNS respiratory sensing lead from January 1, 2000, to December 1, 2022. Primary outcomes were respiratory lead migration resulting in pneumothorax or need for revision surgery/explantation.

**Results:**

In total, 151 out of 765 HGNS adverse events were related to the respiratory sensing lead, and of those, 75 were related to lead migration. There were seven events related to migration of the sensing lead into the pleural space, of which six cases underwent revision surgery (<1% of adverse events reported related to HGNS). Two cases noted pneumothorax due to sensing lead migration. We report two cases of sensing lead migration at our institution. Migration was demonstrated with serial imaging. These cases highlight the potential need for preoperative or intraoperative chest tube placement, based on the extent of migration, complications, and complexity.

**Conclusion:**

Migration of the respiratory sensing lead is a rare event with multidisciplinary surgical planning considerations.

Unilateral hypoglossal nerve stimulation (HGNS) is a second‐line treatment for patients with obstructive sleep apnea (OSA). The only HGNS therapy that has been approved by the United States Food and Drug Administration (FDA) is Upper Airway Stimulation® (UAS; Inspire Medical Systems, Inc.). This system includes three implanted components: an implantable pulse generator placed superficial to the pectoralis muscle, a respiratory sensing lead placed with the tip between the internal and external intercostal muscles, and an HGNS lead whose cuff is placed around a portion of the distal hypoglossal nerve.

For FDA‐approved devices, the Manufacturer and User Facility Device Experience (MAUDE) is a post‐market surveillance database that receives reports of adverse events from device manufacturers, physicians, and patients. Prior studies queried the MAUDE database from 2000 through December 2023 ^1^, ^2^, ^3^, ^4^ and reported rare adverse events that did not occur during the STAR trial or subsequent ADHERE registry publication, including pneumothorax, pleural effusion, and sensing lead migration.^5^, ^6^ A recent publication queried MAUDE from January 2014 to September 2022, focusing on events of sensing lead malfunction or distal tip separation.^3^ However, this study did not focus on migration of the intact sensing lead but rather reported several cases of distal tip separation or malfunction of the sensing lead with emphasis on waveform morphologies. The present paper focuses on the migration of the intact sensing lead.

It is important for physicians to be aware of rare potential adverse events to counsel patients on the risks of HGNS implantation and to inform management. Given the growth in HGNS implantation, an opportunity exists to pool collective experience of this rare adverse event and its management through the use of the MAUDE database. The objective of this study was to provide an updated report of complications from the MAUDE database, with a specific focus on the respiratory sensing lead, and to propose a care algorithm based on our experience with two presented cases of intact sensing lead migration.

## Methods

This research was deemed exempt by the University of Southern California Institutional Review Board. The MAUDE database was queried for events related to the respiratory sensing lead for the HGNS device (code MNQ) for events from January 1, 2000, to December 1, 2022, using the same start date as a previous MAUDE HGNS study.^1^ Following the methods of previous MAUDE HGNS studies, events were reviewed and removed if duplicates, submitted by patients, unrelated to the system or its surgical implantation, based on publications or the Internet alone, or with insufficient information for analysis.^1^

Results from the MAUDE database were filtered to include those events related to the migration of the respiratory sensing lead. All respiratory sensing lead results were manually reviewed to capture events potentially missed or incorrectly coded. Primary outcomes were respiratory sensing lead migration resulting in pneumothorax/pulmonary effusion or need for revision surgery/explantation. Descriptive statistics were calculated for all measures of adverse events and outcomes.

Additionally, two cases performed by the senior author (E.J.K.) of respiratory sensing lead migration are presented.

## Results

### MAUDE Database Review

**Figure 1:**
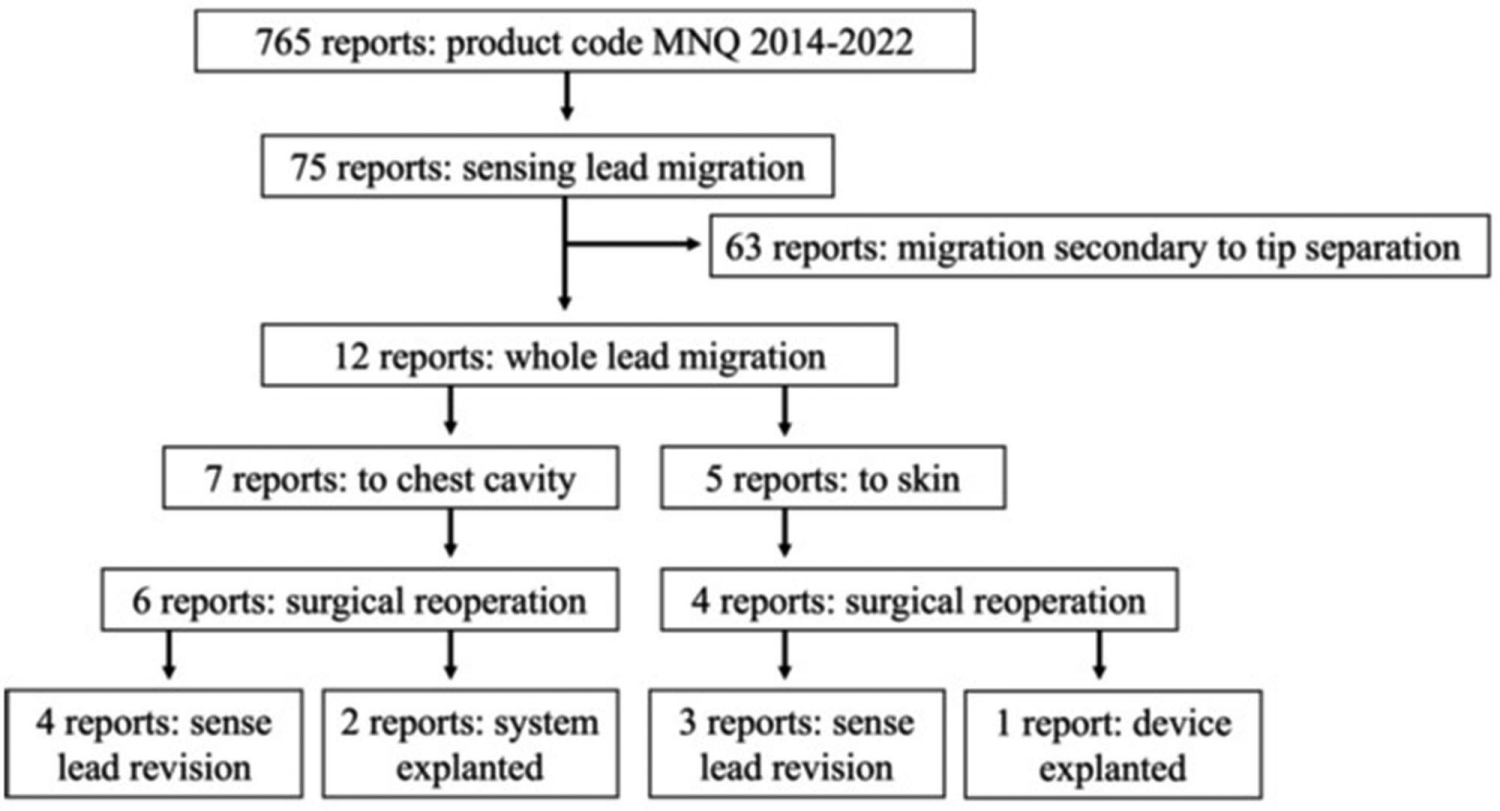
Manufacturer and User Facility Device Experience database review flow chart for hypoglossal nerve stimulator, queried January 2000 to December 2022.

As summarized in Figure 1, 765 adverse events related to device code MNQ were found, with 151 of these related to the respiratory sensing lead. Of these, about 50% (n = 75) were related to respiratory sensing lead migration. The majority (63/75, 84%) included separation of the distal tip of the respiratory sensing lead from the lead body.

Of the 12 adverse events without separation of the distal tip (Table 1), 7 involved migration of the lead into the chest cavity, and 5 had migration superficially towards the skin. Of note, one of the migration events towards the chest occurred in the perioperative period and was revised immediately before discharge. Five cases involved the respiratory sensing lead model 4340; the remainder involved model 4323. The 4340 model sensing lead is a newer, redesigned model intended to reduce the risk of tip separation or migration.^6^ Of the seven chest cavity migration cases, four underwent revision surgery, and two were explanted. Two cases reported pneumothorax and chest tube placement (one in the perioperative period and one delayed). Of the two cases with pneumothorax, one patient underwent revision, and the other explanation. Common documented symptoms included pain, dyspnea, and/or malfunction of the device (respiratory sensing signal diminished). For migration events towards the skin, wound dehiscence was noted in all cases.

**Table 1:** Manufacturer and User Facility Device Experience Database Adverse Events Related to Hypoglossal Nerve Stimulator (Code MNQ) Migration of an Intact Respiratory Sensing Lead, Years 2000 to 2022^a^

Case	Year	Sensor model	Occurrence	Migration location	Symptoms	Treatment^b^
**1**	**2015**	**4323**	**Delayed**	**Chest cavity/pleura**	**Malfunction**	**Explanted**
**2**	**2019**	**4323**	**Delayed**	**Chest cavity/pleura**	**Pain**	**None/unknown**
3	2019	4323	Delayed	Skin	Dehiscence	None/unknown
**4**	**2020**	**4340**	**Immediate**	**Chest cavity/pleura**	**Pneumothorax**	**Repositioned, chest tube**
**5**	**2020**	**4323**	**Delayed**	**Chest cavity/pleura**	**Lump**	**Replaced**
6	2020	4340	Delayed	Skin	Dehiscence	Removed
**7**	**2021**	**4323**	**Delayed**	**Chest cavity/pleura**	**Pain**	**Replaced**
**8**	**2021**	**4323**	**Delayed**	**Chest cavity/pleura**	**Dyspnea**	**Replaced**
9	2021	4340	Delayed	Skin	Dehiscence	Removal, antibiotics (oral)
10	2021	4340	Delayed	Skin	Infection, dehiscence	Explanted, antibiotics (IV)
11	2021	4340	Delayed	Skin	Dehiscence	Replaced
**12**	**2022**	**4323**	**Delayed**	**Chest cavity/pleura**	**Pain, pneumothorax**	**Explanted, chest tube**

Abbreviation: IV, intravenous.

^a^ Cases related to migration into the chest cavity/pleura are bolded.

^b^ Explanted indicates the entire system was removed. Repositioned, replaced, and removed refer to the respiratory sensing lead.

### Case Report

The following cases are included in the MAUDE database findings above.

#### Case 1

**Figure 2:**
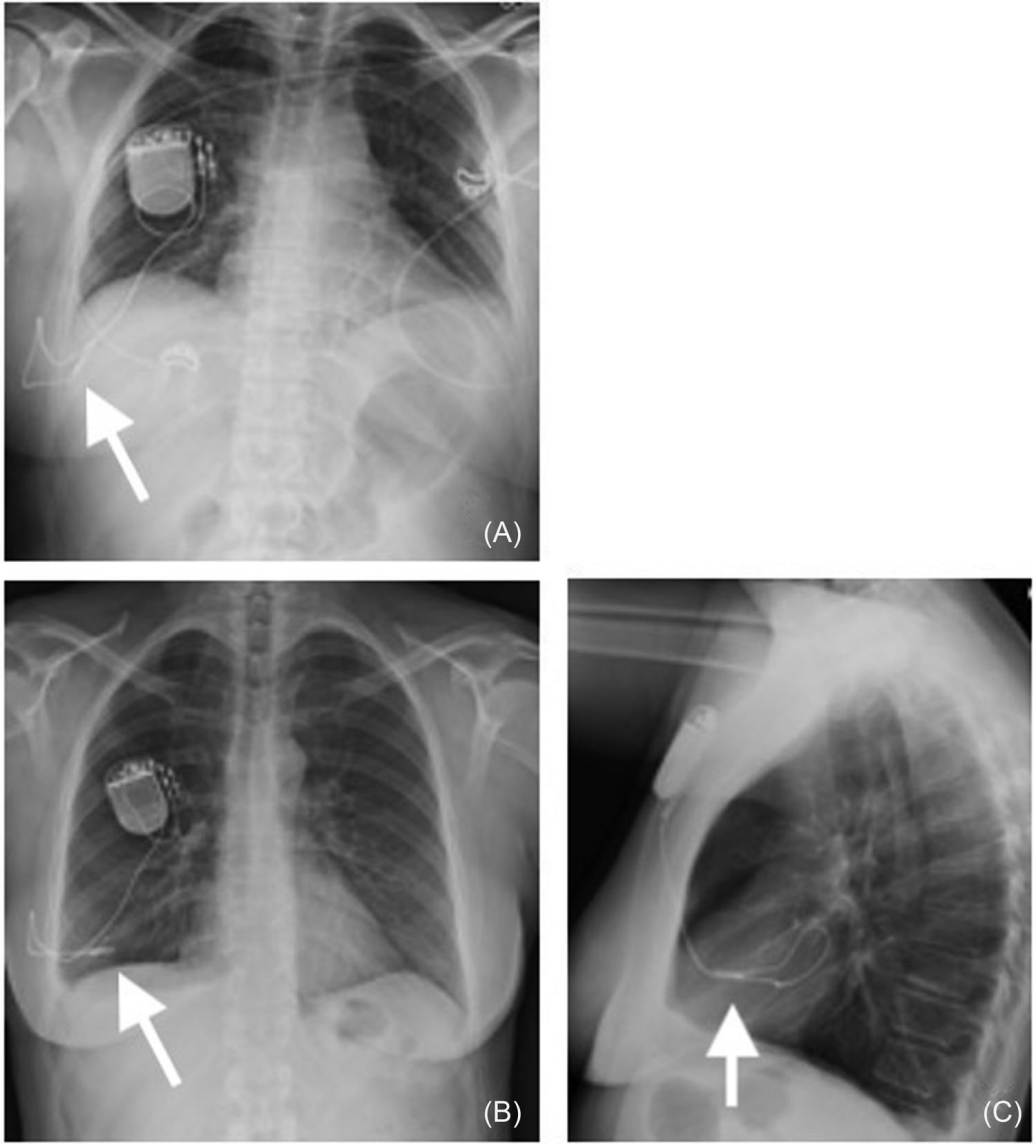
Case 1 migration of hypoglossal nerve stimulator respiratory sensing lead with immediate postoperative anterior‐posterior chest x‐ray (A) and x‐rays at time of migration—anterior‐posterior (B) and lateral (C) views. The arrow indicates the respiratory sensing lead location.

A 52‐year‐old Caucasian female, a never‐smoker, was implanted with an HGNS device via a three‐incision approach. Her preoperative body mass index (BMI) and apnea‐hypopnea index (AHI) were 29.5 kg/m^2^ and 26.8 events/hour, respectively. No technical difficulties were noted for respiratory sensor insertion at the time of initial operation. Immediate postoperative x‐rays showed good placement of the electrode and respiratory sensing lead with no effusion or pneumothorax. The initial activation range was 0.9 to 1.9 V with an electrode configuration of plus‐minus‐plus. Postoperative titration study demonstrated an AHI of 5.4 at 2 V, capturing REM and supine sleep. OSA symptoms improved dramatically. Eight months postoperatively, the patient developed moderate to severe right‐sided chest pain overlying the respiratory sensing lead, which radiated to the right axilla and worsened with deep inspiration and arm movement. Workup at that time, including device interrogation, chest x‐rays, EKG, and echocardiogram, was negative with no changes in impedance or waveform of the device noted. The symptoms resolved. Thirty‐two months postoperatively, the chest pain recurred at a similar intensity, and the patient responded to gabapentin and celecoxib. Voltage requirements increased to 3.7 to 4.7 V. During this time, the patient's BMI decreased from 28 to 25 kg/m^2^. Chest pain episodes occurred intermittently until approximately 36 months postoperatively, when the pain became constant and there was a noted increase in snoring and other OSA symptoms as well as a weak sensation of tongue movement with stimulation despite no changes in system settings. Chest x‐rays at that time showed pleural effusion and migration of the respiratory sensing lead into the pleural space (Figure 2). The patient underwent revision surgery, where preoperative impedance testing was consistent with malfunction of the respiratory sensing lead. The respiratory sensing lead was removed at the initial site of entry within the fifth intercostal space, and a new lead was placed in the second intercostal space. There was no apparent migration of the sensing lead at the initial entry between the internal and external intercostal muscles. Postoperatively, the patient was reactivated with a range of 0.8 to 1.6 V in off‐minus‐off configuration with resolution of chest pain and OSA symptoms. Home sleep apnea test following replacement/revision showed AHI of 8.8. The patient was followed for 1 year after revision at the time of chart review.

#### Case 2

**Figure 3:**
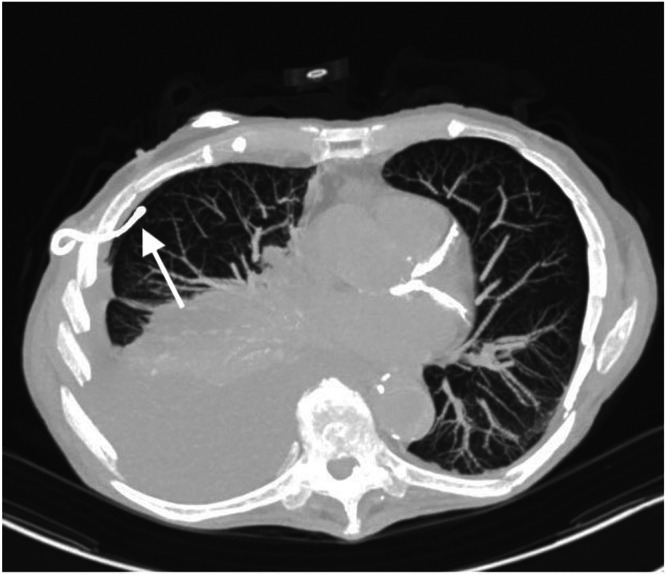
Case 2 axial computed tomography scan image showing hypoglossal nerve stimulator respiratory sensing lead intrapleural. The arrow indicates the respiratory sensing lead location.

An 84‐year‐old Caucasian male, never smoker, was implanted via a three‐incision approach with preoperative BMI and AHI of 20.6 kg/m^2^ and 27 events/hour, respectively. No technical difficulties were noted for respiratory sensor insertion at the time of the initial operation, and postoperative x‐ray did not show pleural effusion or pneumothorax. Post implantation, the patient had initial difficulty tolerating stimulation at therapeutic range and achieving adequate sleep time due to comorbid difficulty initiating and maintaining sleep. Postoperative titration study 2 months post implantation showed good control of AHI with the recommended range of 1.7 to 2.1 V, with REM sleep observed. He intermittently used the system. The patient was lost to follow‐up for 3 years until he presented to the hospital with fatigue and dyspnea. Primary care provider documentation noted bilateral pneumonia on outside chest x‐ray in the weeks preceding hospital admission. At the time of admission, chest computed tomography showed hydropneumothorax and migration of the respiratory sensing lead into the pleural space (Figure 3). Imaging also revealed bilateral pulmonary nodules of indeterminate significance that were felt to be unrelated to the migration. A chest tube was placed with pleural fluid showing no malignant cells. As the patient was not using therapy, explantation was planned following hospital admission. Given the location of the respiratory sensing lead into the pleural space, thoracic surgery was preemptively notified and available on the day of explantation. There was no migration of the sensing lead out of the original rib space. Respiratory sensing lead was carefully dissected between intercostal muscles to minimize any additional opening into the pleural space, but immediately following removal of the respiratory sensing lead, air bubbles were noted within the chest field and on subsequent breaths. Occlusive pressure was applied, and the thoracic surgery team was called into the operating room for placement of a 28 Fr straight chest tube and wound closure in layers. The patient was observed postoperatively overnight, with serial x‐rays showing reduced size of apical pneumothorax and small right pleural effusion. The patient was discharged on postoperative day 1 after removal of the chest tube. In the months post explant, the patient had recurrences of hydropneumothorax that were treated with placement of pigtail chest tubes. The patient was followed for 2 months after surgery.

## Discussion

This is the first HGNS study to focus on events of migration of the intact respiratory sensing lead, specifically, incorporating an algorithm for evaluation of possible sensing lead migration. We also explore the incidence of pneumothorax as it relates to migration of an intact respiratory sensing lead.^1^, ^2^, ^5^

Multiple publications have utilized the MAUDE database, including two recent ones from the same group.^3^, ^4^ Our paper offers an in‐depth analysis of the intact sensing lead migration events through 2022 paired with a report of two cases for greater context. Differences among these papers can be accounted for by differences in methodology and the timeframe studied. The present study makes the transition from reporting and analyzing the source of MAUDE events to incorporating the information into clinical practice, proposing an evaluation and treatment algorithm (discussed below).

Distinguishing between the two types of respiratory sensing lead migration (intact lead vs distal tip separation) is important, as distal tip separation is a known occurrence and has been addressed by the company with modification of technique with utilization of specific “gripping zone” for manipulating lead with gentle pressure and design of the respiratory sensing lead. All implanting surgeons undergo required training addressing the handling of the lead to reduce the risk of separation. The new respiratory sensing lead model 4340 sensing lead was approved by the FDA in 2019, designed with a slimmer shape and to minimize tip separation.^7^ In cases of migration of the lead into the pleural cavity and distal tip separation, removal of the distal tip itself generally requires multidisciplinary management with thoracic surgery.

**Figure 4:**
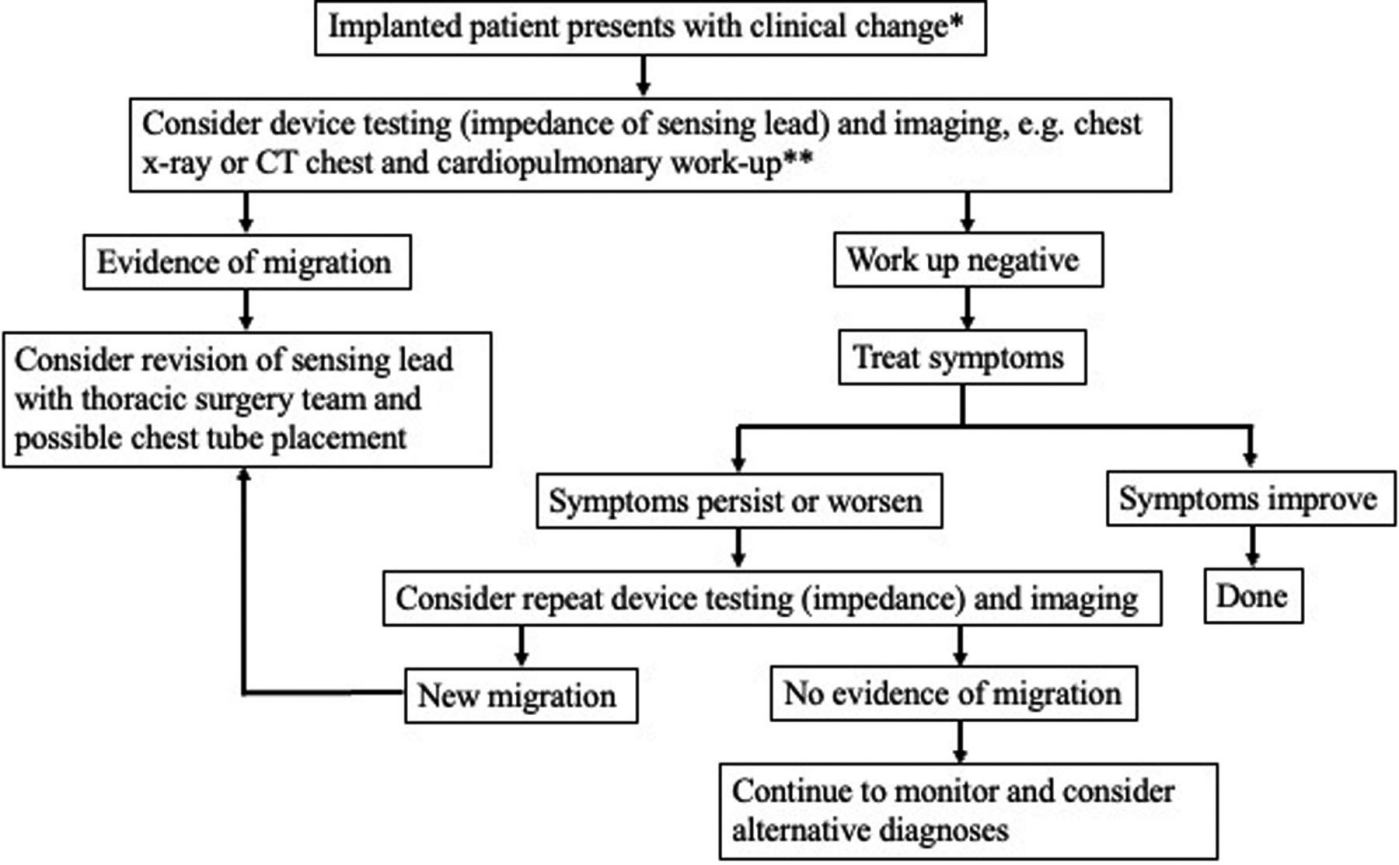
Decision tree for management of suspected hypoglossal nerve stimulator respiratory sensing lead migration. *Clinical change may include, but not limited to, new chest pain, shortness of breath, and change in voltage requirements. **Cardiopulmonary workup may include, but not limited to, electrocardiogram, echocardiogram, lab tests, and multidisciplinary consultation. CT, computed tomography.

Migration of an intact respiratory sensing lead is rare, with 12 reports found in the MAUDE database from 2000 to 2022 (effectively 2014‐2022 based on device initial FDA approval). This study provides a broader clinical experience to this distinct entity, with intervention and symptom pattern emergence. Reoperation was common, with all cases requiring reoperation except for one case of skin migration. Our study shows symptom patterns emerging among patients with migration of the respiratory sensing lead to include pain, dyspnea, and change in device function. Based on our experience, we have proposed a decision tree (Figure 4) that focuses on symptom monitoring, consideration of repeat imaging, and multidisciplinary planning. In the senior author's practice, the HGNS device is interrogated at all follow‐up visits (typically every 6 months once the patient is stable on treatment, with good clinical response, and then annually if stable at 2 years post implant). Additional visits and interrogation of the device occur if any issues. Figure 4 is intended for a clinical change deviating from normal follow‐up.

Case 1 of the case report further highlights this symptom constellation and clinical findings for pleural migration with chest pain, abrupt change in sensation of tongue stimulation, and increasing therapeutic voltage requirements. When symptoms arise or HGNS functioning changes, repeat imaging may be indicated (even if prior imaging was normal).

### Postoperative Pneumothorax

Pneumothorax associated with HGNS can occur at the time of surgery (often related to placement of the sensing lead) or in a delayed fashion, potentially related to migration of the sensing lead or a portion of the sensing lead tip. In our study, only two of the seven chest cavity migration events were associated with reported pneumothorax, both requiring chest tube placement (0.2% of MAUDE reported events for the HGNS device). Prior literature reports indicate that 4% of adverse events (50/1312) report pneumothorax in the MAUDE database between 2000 and 2023.^4^ Another study looking at rates of pneumothorax following HGNS implantation between sleep surgeons and general otolaryngologists noted a pneumothorax rate of 3.4% for general otolaryngologist and 0.21% for sleep surgeons in a sample size of 1233 subjects undergoing HGNS implantation; with greater than 90% of the pneumothoraxes occurring within 1 day of implantation.^8^ Including pleural effusions, another study using the TriNetX network cited a 2.4% pneumothorax rate and 0.6% pleural effusion rate in the 1813 patient cohort with noted increased rate in patients with chronic lower respiratory disease.^9^

To our knowledge, there are no other studies reporting delayed pneumothorax with migration of the respiratory sensing lead as a potential associated cause or risk factor. As routine serial postoperative imaging is not performed without symptoms, the incidence of respiratory sensing lead migration is unknown. With case 2 of our series, it is unclear if the delayed hydropneumothorax was caused by respiratory sensing lead migration, as the patient had subsequent recurrences following explantation. This patient was thin with a BMI less than 21 but otherwise did not have known risk factors for spontaneous pneumothorax.^10^

### Decision to Convert to Two‐Incision Approach

Two‐incision implantation of HGNS has become the preferred technique due to its ease of technique and reduced surgical time.^11^ In case 1, we opted to remove the existing sensing lead entirely from the fifth intercostal space and place a new sensing lead in the second intercostal space. This move offered placement in a previously unoperated area with improved access for any potential future revision. Further, design adjustments with shortening of the respiratory sensing lead wire make a shared surgical site more conducive.

### Limitations

The major limitations of this study are due to limitations of the MAUDE database itself. There may be bias in reporting only certain types of events. Given the mandatory reporting guidelines and clinically apparent sequelae of respiratory sensing lead migration into the pleural space (eg, system malfunction, pleural effusion, and pneumothorax), unreported events of this nature are less likely. The database does not contain patient information including age, gender/sex, race, BMI, and comorbidities that may influence adverse event rates, so it was not possible to study these risk factors. Additionally, the date of initial implantation is not included, and therefore, the time course over which migration may have occurred is unclear. The technique of operation (two‐ vs three‐incision technique) is also unknown. Regarding the case report, this information is limited by the retrospective nature of the data collection and is isolated to the findings of one institution and surgeon. Given the small sample size and limited data, the investigation into the mechanism of migration is limited.

Use of the MAUDE database increases the generalizability of the findings, and, supplemented by the case report, greater context can be given to these rare events of migration of an intact respiratory sensing lead.

## Conclusion

Migration of the intact HGNS respiratory sensing lead is a rare event, with migration into the chest cavity accounting for less than 1% of events related to HGNS reported to the MAUDE database. As intrathoracic migration does not present with visual surgical site changes, clinical picture and associated symptoms including dyspnea, surgical site pain, and device malfunction may be important clues. A multidisciplinary approach and coordination with thoracic surgery may be important for surgical planning. Future research including patient medical history and demographic information is needed to investigate potential risk factors for respiratory sensing lead migration as well as research investigating mechanisms and causes of migration. Additionally, further studies are needed to investigate the association between surgeon experience and respiratory sensing lead migration.

## Author Contributions

**Emily A. Commesso**, Design; conduct; analysis; presentation of the research. **Marcus F. Paoletti**, Conduct; analysis; presentation of the research. **Eric J. Kezirian**, Design; analysis; presentation of the research.

## Disclosures

### Competing interests

Emily A. Commesso discloses that she is a consultant for Guidepoint. Eric J. Kezirian discloses that he is a consultant for Berendo Scientific, Cryosa, huMannity Medtec, Nyxoah, and Split Rock Scientific. He was a clinical trial investigator for Inspire Medical Systems. He owns intellectual property with Berendo Scientific and Magnap.

### Funding source

None.
